# Protective Effects of Maclurin against Benzo[a]pyrene via Aryl Hydrocarbon Receptor and Nuclear Factor Erythroid 2-Related Factor 2 Targeting

**DOI:** 10.3390/antiox10081189

**Published:** 2021-07-26

**Authors:** Jangsoon Kim, See-Hyoung Park, Seyoung Yang, Sae Woong Oh, Kitae Kwon, Se Jung Park, Eunbi Yu, Hyeyoun Kim, Jung Yoen Park, Seoyoung Choi, Seoyeon Yang, Minkyung Song, Jae Youl Cho, Jongsung Lee

**Affiliations:** 1 Molecular Dermatology Laboratory, Department of Integrative Biotechnology, College of Biotechnology and Bioengineering, Sungkyunkwan University, Suwon City 16419, Gyunggi do, Korea; rovett@skku.edu (J.K.); yy1771@skku.edu (S.Y.); hanzeeoo@skku.edu (S.W.O.); wesdwe1@skku.edu (K.K.); tpwjd17@skku.edu (S.J.P.); yuebi95@skku.edu (E.Y.); yeon6389@skku.edu (H.K.); maria0502@skku.edu (J.Y.P.); csy2696@skku.edu (S.C.); chorim1004@skku.edu (S.Y.); 2 Department of Bio and Chemical Engineering, Hongik University, Sejong City 30016, Korea; shpark74@hongik.ac.kr; 3 T Cell and Tumor Immunology Laboratory, Department of Integrative Biotechnology, College of Biotechnology and Bioengineering, Sungkyunkwan University, Suwon City 16419, Gyunggi do, Korea; 4 Molecular Immunology Laboratory, Department of Integrative Biotechnology, College of Biotechnology and Bioengineering, Sungkyunkwan University, Suwon City 16419, Gyunggi do, Korea

**Keywords:** maclurin, benzo[a]pyrene, aryl hydrocarbon receptor (AHR), oxidative stress, nuclear factor (erythroid-derived 2)-like 2 (Nrf2), p38 MAPK

## Abstract

Benzo[a]pyrene (B[a]P), a polycyclic aromatic hydrocarbon formed during the incomplete combustion of organic matter, has harmful effects. Therefore, much research is ongoing to develop agents that can mitigate the effects of B[a]P. The aim of this study was to examine the effect of maclurin, one component of the branches of *Morus alba* L., on the B[a]P-induced effects in HaCaT cells, a human keratinocyte cell line. Maclurin treatment inhibited aryl hydrocarbon receptor (AHR) signaling as evidenced by reduced xenobiotic response element (XRE) reporter activity, decreased expression of cytochrome P450 1A1 (CYP1A1), and reduced nuclear translocation of AHR. The B[a]P-induced dissociation of AHR from AHR-interacting protein (AIP) was suppressed by maclurin. Maclurin also inhibited the production of intracellular reactive oxygen species (ROS) induced by B[a]P. In addition, the antioxidant property of maclurin itself was demonstrated by the 2,2-diphenyl-1-picrylhydrazyl (DPPH) radical scavenging assay. Furthermore, maclurin activated antioxidant response element (ARE) signaling through enhancement of ARE luciferase reporter activity and the expression of ARE-dependent genes including nuclear factor (erythroid-derived 2)-like 2 (Nrf2) and heme oxygenase-1 (HO-1). Nrf2 activation and its nuclear translocation were promoted by maclurin through p38 MAPK activation. These data indicate that maclurin had antagonistic activity against B[a]P effects through activation of Nrf2-mediated signaling and inhibition of AHR signaling and, suggesting its potential in protecting from harmful B[a]P-containing pollutants.

## 1. Introduction

Air pollution is becoming more serious every day. Many types of air pollutants negatively affect our health and daily life. One of the well-known air pollutants is a benzo[a]pyrene (B[a]P). B[a]P is generated by incomplete combustion and can be found in automobile exhaust fumes, cigarette smoke, charbroiled food, emissions from residential wood burning, and volcanoes [1,2,3].

B[a]P can be absorbed via several routes including the oral route, inhalation, and epidermal exposure [4,5,6]. Since the skin covers the surface of the entire body, it can become one of the main routes for B[a]P penetration into the body. Among the cell types in the epidermis, the outermost layer of the skin, the keratinocytes, are present in the largest number. Therefore, keratinocytes have a pivotal role in sensing and reacting to environmental stimuli.

In addition, since B[a]P has a hydrophobic property as a type of polycyclic aromatic hydrocarbon, it can penetrate cell membranes. When B[a]P enters the cell, many enzymes work to release the B[a]P by increasing the water solubility of B[a]P and generating a large number of B[a]P metabolites [7]. Among them, (±)-anti-benzo[a]pyrene-7,8-diol-9,10-epoxide (BPDE) is a well-known carcinogenic intermediate and can form DNA adducts. Cytochrome P4501A1 (CYP1A1) plays a key role in the formation of BPDE [8] and because CYP1A1 is a type of monooxygenase, it can induce the generation of reactive oxygen species (ROS) [7].

CYP1A1 expression is regulated by the aryl hydrocarbon receptor (AHR) [9]. When B[a]P attaches to the AHR, the B[a]P-AHR complex moves to the nucleus. Following nuclear translocation, this complex combines with AHR nuclear translocator (ARNT) [10]. Lastly, the complex interacts with xenobiotic response elements (XREs) and induces the expression of target genes like CYP1A1 [11]. 

One of the major regulators of oxidative stress in cells is nuclear factor erythroid 2-related factor 2 (Nrf2) [12]. Nrf2, a transcription factor, regulates anti-oxidant enzymes such as heme oxygenase-1 (HO-1) and the others via antioxidant responsive element (ARE) recognition [13]. Nrf2 is present in the cytoplasm with Kelch-like ECH-associated protein 1 (Keap1) and Cullin 3 (Cul3). Keap1 and Cul3 induce the decomposition of Nrf2. However, under oxidative stress conditions, the Nrf2-keap1-cul3 complex is broken down. Free Nrf2 moves to the nucleus and binds to the ARE sequence, inducing the expression of target genes [12,14,15,16].

**Figure 1:**
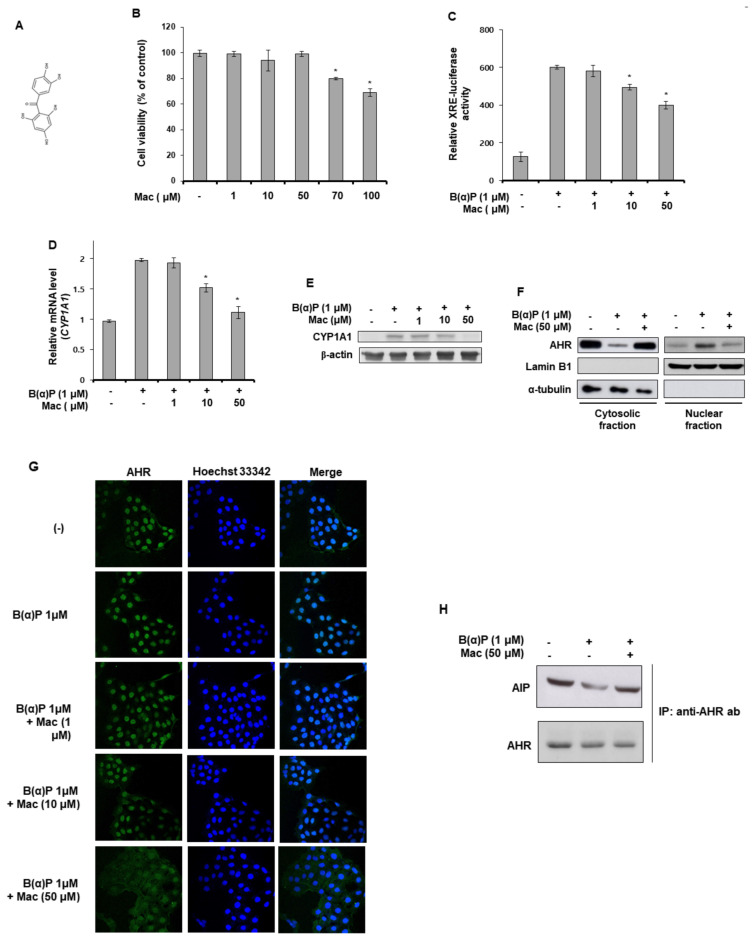
Maclurin suppressed B(a)P-induced aromatic hydrocarbon receptor (AHR) signaling in HaCaT cells. (**A**) Chemical structure of maclurin. (**B**) HaCaT cells were incubated with maclurin. After 24 h of incubation, cells were subjected to cell viability assay. Data are presented as mean ± SD of three replicates. * *p* < 0.05 versus control group. (**C**) The DharmaFECT^®^ Duo transfection reagent was used to transfect HaCaT cells with the XRE-Luc reporter together with a Renilla-luciferase vector. After 24 h incubation, the cells were incubated with maclurin and B(a)P under serum-free conditions for 14 h. The cells were lysed and analyzed by the luciferase reporter assay. The data are presented as the mean ± SD of three replicates. * *p* < 0.05 versus B(a)P treatment group. (**D**) The cells were treated with B(a)P in the presence or absence of maclurin. After 24 h, the cells were harvested and analyzed by RT-PCR (**D**) and Western blots (**E**) for CYP1A1. The data are presented as the mean ± SD of three replicates. * *p* < 0.05 versus B(a)P treatment group. (**F**,**G**) HaCaT cells were treated with maclurin and B(a)P for 24 h and analyzed by Western blotting (**F**) of the cytosolic and nuclear fractions and immunocytochemistry (original magnification: 20X) (**G**) to assess AHR translocation. (**H**) HaCaT cells were treated with maclurin and B(a)P for 24 h and analyzed by immunoprecipitation with anti-AHR antibodies and Western blotting of AIP and AHR. Mac: maclurin.

Various reports have demonstrated the cytotoxic, carcinogenic, oxidative stress, and mutagenic effects of B[a]P [17,18,19,20]. However, until now, there have been only a few reports regarding the development of compounds to reduce the toxicity of B[a]P [21,22]. Maclurin ((3,4-dihydroxyphenyl)-(2,4,6-trihydroxyphenyl) methanone) (Figure 1A) is the target molecule of this study. Maclurin is found in *Morus alba* (white mulberry) and *Garcinia mangostana* (purple mangosteen) and several researchers have reported its anti-cancer and anti-oxidant properties. [23,24]. In addition, except for a report from our group on the melanogenic property of maclurin, maclurin has not been elucidated to be involved in skin cell biology. Specifically, previous studies have not reported its protective effects against B[a]P and the signal transduction pathways involved in human keratinocytes.

In this study, we examined the effect of maclurin on the B[a]P-induced effects in human keratinocytes and its action mechanism.

## 2. Materials and Methods

### 2.1. Materials and Reagents 

Maclurin (purity: 99%) was acquired from Chirochem (Daejeon, Korea) and was dissolved in dimethyl sulfoxide (DMSO). The concentration of maclurin stock solution was 50 mM. LaminB1 and α-tubulin antibodies were acquired from Epitomic (Epitomic, Burlingame, CA, USA). Dulbecco’s modified Eagle’s medium (DMEM), vitamin C, and trypan blue were purchased from Thermo Fisher Scientific, Inc. (Waltham, MA, USA). B[a]P, DPPH, and 2,2′-azino-bis(3-ethylbenzothiazoline-6-sulfonic acid) (ABTS) were obtained from Sigma (Sigma-Aldrich Co., St. Louis, MO, USA) AHR, CYP1A1, and HO-1 antibodies were obtained from Santa Cruz (Santa Cruz Biotechnology, Santa Cruz, CA, USA). The concentration of B[a]P stock solution was 1 mM.

### 2.2. Culture of Cells 

HaCaT cells (American Type Culture Collection, Manassas, VA, USA), a human keratinocyte cell line was maintained in a humidified 5% CO_2_ atmosphere at 37 °C and cultured in DMEM supplemented with 10% fetal bovine serum (FBS) and 1% antibiotics (penicillin/streptomycin).

### 2.3. Assay of Cell Viability Assay 

The effect of maclurin on cell viability was measured using a cell counting kit-8 (CCK-8, Dojindo, Japan). HaCaT cells were cultured in 6-well plates and treated with indicated concentrations (1, 10, 50, 70, and 100 μM) of maclurin. After 24 h treatment, CCK-8 (8 μL/well) was added and the plates were incubated in a humidified 5% CO_2_ atmosphere at 37 °C for 2 h. The cell supernatant was added to a 96-well plate, and the absorbance was determined at 450 nm using a microplate reader (Synergy HTX Multi-Mode Reader, Biotek, Winooski, VT, USA). 

### 2.4. Measurement of XRE and ARE Activity 

HaCaT cells were cultured in 6-well plates prior to transient transfection with XRE (Stratagene, La Jolla, CA, USA) or an ARE-driven luciferase reporter plasmid (1.0 μg each well), (Addgene, Watertown, MA, USA) along with β-galactosidase in polyethyleneimine (PEI) (Sigma-Aldrich). After 4 h, the medium was replaced with fresh medium for stabilization. The transfected cells were treated with maclurin (1, 10, 50 μM) in the presence or absence of 1 μM B[a]P for 24 h. The cells were harvested by scraping and centrifuged. The cells were lysed with Reporter Lysis Buffer and centrifuged. The luciferase and β-galactosidase activity in the supernatants were measured according to the manufacturer’s instructions (Promega Corporation, Madison, WI, USA). The final activity was displayed as the ratio of luciferase activity to β-galactosidase activity.

### 2.5. BPDE-DNA Adduct Formation Analysis 

HaCaT cells in DMEM were seeded into 6-well plates and then incubated for 24 h. After 24 h of incubation, cells were treated with 1 μM B[a]P in the presence or absence of maclurin for 72 h. DNA was extracted from the HaCaT cells at the end of the treatment period using the QIAamp DNA Mini Kit (Qiagen, Stanford, CA, USA) according to the manufacturer’s instructions. The isolated DNA was analyzed for BPDE-DNA adduct formation using a BPDE-DNA adduct ELISA kit (Cell Biolabs, San Diego, CA, USA) according to the manufacturer’s instructions. The relative BPDE-DNA adduct levels were measured using a microplate reader at 450 nm absorbance.

### 2.6. Western Blot Analysis 

HaCaT cells were seeded in 60 mm dish plates. The cells were treated with maclurin (1, 10, 50 μM) and 1 μM B[a]P. To prepare the samples for Western blots, the cells were harvested and centrifuged. The cell pellets were lysed by RIPA lysis buffer [150 mM NaCl, 25 mM Tris-HCl (pH 7.6), 1% sodium deoxycholate, 1% NP-40, 0.1% SDS (Thermo Fisher Scientific)] including protease and phosphatase inhibitor (Thermo Fisher Scientific). For the preparation of cytoplasmic and nuclear fractions, NE-PER Nuclear and Cytoplasmic Extraction reagents (78833, Thermo Fisher Scientific) were employed according to the manufacturer’s protocol. The protein content in the samples was quantified with a BCA assay kit (Thermo Fisher Scientific) and separated by sodium dodecyl sulfate-polyacrylamide gel electrophoresis. The proteins were transferred to polyvinylidene difluoride membranes, blocked, and exposed to the antibodies. Finally, the proteins were detected using ECL Western Blotting Reagents (Thermo Fisher Scientific).

### 2.7. DCFDA Cellular Reactive Oxygen Species Detection 

To determine if B[a]P and maclurin changed the amount of ROS, a DCFDA Cellular Reactive Oxygen Species (ROS) Detection Assay Kit (ab113851, Abcam, Cambridge, UK) was used. The cells were seeded in a 96-well plate and incubated with maclurin (1, 10, 50 μM) in the presence or absence of 1 μM B[a]P. tert-Butyl hydroperoxide (TBHP) was used as a positive control. Next, the cultured cells were stained with 20 µM 5(6)-Carboxy-2′,7′-dichlorofluorescein diacetate (DCFDA) in phosphate-buffered saline PBS for 15 min at 37 °C in the dark. After washing the cells, the fluorescence intensity was detected at excitation and emission wavelengths of 485 and 535 nm, respectively.

### 2.8. DPPH Radical Scavenging Assay 

The radical scavenging activity of maclurin was analyzed using DPPH. The final maclurin (1, 10, 50 μM) and ascorbic acid (100 μM) solutions were made in 0.15 mM DPPH. The absorbance was measured at 517 nm. Vitamin C (Vit C) was introduced as a positive control. The percentage of radical scavenging was measured using the equation:

% radical scavenging activity = [(Absorbance of control − Absorbance of sample)/Absorbance of control] × 100

### 2.9. ABTS Radical Scavenging Assay 

The ABTS radical was prepared by reacting 14 mM ABTS and 4.9 mM potassium persulfate in the dark for 12–16 h at room temperature. Before assaying the scavenging assay, the ABTS radical was diluted with PBS to obtain an initial absorbance of 0.7 ± 0.02 at 734 nm. The final maclurin (1, 10, 50 μM) and ascorbic acid (100 μM) solutions were made in the ABTS radical solution. Next, the absorbance was measured at 734 nm. Vit C was introduced as a positive control Lastly, the radical scavenging activity was determined using the equation in Section 2.8.

### 2.10. Immunocytochemistry 

Immunocytochemistry analysis was conducted as previously described [25]. Specifically, while for fixation, cells were treated with 4% paraformaldehyde in PBS for 15 min, they were incubated with 0.1% Triton X-100 and 0.01% Tween-20 for 20 min at room temperature for the permeabilization. As a next step, cells were blocked with PBS containing 3% bovine serum albumin (BSA), and were then incubated with anti-AHR (1:500; Cell Signaling Technology) antibodies. After washing three times, they were treated with Flamma-488 secondary antibodies (Bioacts, Incheon, Republic of Korea). Finally, the cells were mounted on glass slides after counterstaining with 4′,6-diamidino-2-phenylindole (DAPI) and observed under an LSM 700 laser scanning confocal microscope (Zeiss, Jena, Germany) with a C-Apochromat 20× objective. For the measurement of immunofluorescence intensity, the images were captured at the same laser power and the mean intensity of the fluorescence signals was measured. The data were analyzed using ZEN 2012 Blue (Zeiss) and ImageJ1.52a software (National Institutes of Health, Bethesda, MD, USA), under the same processing parameters.

### 2.11. Small-Interfering RNA (siRNA) for Nrf2 and AHR 

ON-TARGETplus SMARTpool human Nrf2 siRNA (L-004018-00-0020), ON-TARGETplus SMARTpool human AHR siRNA (L-004990-00-0020), and ON-TARGETplus non-targeting siRNA (D-001810-10-05) were obtained from Thermo Fisher Scientific, Inc. The cells were transfected with the indicated siRNAs at 50 nM for 24 h using the DharmaFECT transfection agent (Dharmacon Research, Lafayette, CO, USA) according to the manufacturer’s instructions.

### 2.12. Analysis of mRNA Levels Using Real-Time PCR 

Real-time polymerase chain reaction (RT-PCR) analysis was conducted as previously described [25]. Specifically, RT-PCR analysis was done with an ABI7900HT Instrument (Applied Biosystems, Waltham, MA, USA). Predesigned or optimized assays on demand (Applied Biosystems) were used for TaqMan analysis. They include Nrf2 (ID: Hs00975961_g1), CYP1A1 (ID: Hs01054796_g1), HO-1 (ID: Hs01110250_m1), glyceraldehyde-3-phosphate dehydrogenase (GAPDH) (ID: Hs00266705_g1), 18S (Hs03003631_g1), and hypoxanthine-guanine phosphoribosyltransferase (HPRT) (Hs02800695_m1). ABI Sequence Detector Software version 2.0 (Applied Biosystems, Waltham, MA, USA) was used to analyze the data. TRI reagent^®^ was introduced to extract total RNA from the cells according to the manufacturer’s protocols and the total RNA was stored at –70 °C until use. MuLV reverse transcriptase was used to synthesize cDNA from the total RNA (1 μg) according to the manufacturer’s instructions. RT-PCR results was analyzed as previously reported [19]. The data were normalized to the expression level of three housekeeping genes such as GAPDH, 18S, and HPRT. The expression levels of target genes were normalized to the levels measured in the controls. The same experiment was repeated four times to verify the results and each experiment was conducted in triplicate. 

### 2.13. Enzyme-Linked Immunosorbent Assay (ELISA) 

ELISA analysis was conducted as previously described [26]. Specifically, an IL-8 ELISA Kit (Invitrogen, Carlsbad, CA, USA) was used to measure the levels of IL-8 according to the manufacturer’s protocol. The absorbance was measured using a Labsystems Multiskan MS Analyzer (Thermo Bio-Analysis Japan, Tokyo, Japan). The results were confirmed by repeating the same experiment three times.

### 2.14. Statistical Analysis 

All results are expressed as the mean ± standard deviation of at least three experiments. A Student’s *t*-test for the independent samples was used for the statistical analysis of the data. Values of * *p* < 0.05 displayed statistical significance.

## 3. Results

### 3.1. Maclurin Suppresses B[a]P-Induced AHR Sgnaling and Expression of the CYP1A1 Gene

B(a)P activates AHR protein, which recognizes XRE, inducing the expression of the CYP1A1 gene. Therefore, we investigated the involvement of maclurin (Figure 1A) in B(a)P-induced signaling using the XRE-luciferase reporter assay, RT-PCR analysis, and Western blot analysis. As shown in Figure 1B, the B(a)P-induced XRE reporter activation was reduced by maclurin treatment in a concentration-dependent manner. The CYP1A1 mRNA and protein levels increased by B[a]P treatment were decreased by maclurin treatment (Figure 1C,D). In addition, maclurin treatment suppressed the nuclear translocation of AHR induced by B(a)P, as evidenced by Western blot analysis (Figure 1E) and immunocytochemistry assays (Figure 1F). Furthermore, in the immunoprecipitation experiment with anti-AHR antibodies to elucidate the possible action mechanism of maclurin in the nuclear translocation of AHR, while B(a)P induced the dissociation of AHR from the AHR-interacting protein (AIP) complex, maclurin treatment suppressed it (Figure 1G). These data indicate that maclurin attenuated the AHR signaling induced B[a]P by suppressing the dissociation of the AHR-AIP complex.

### 3.2. Maclurin Attenuates B(a)P Effects on the Intracellular Production of ROS and IL-8, Cell Survival, and BPDE-DNA Adduct Formation

**Figure 2:**
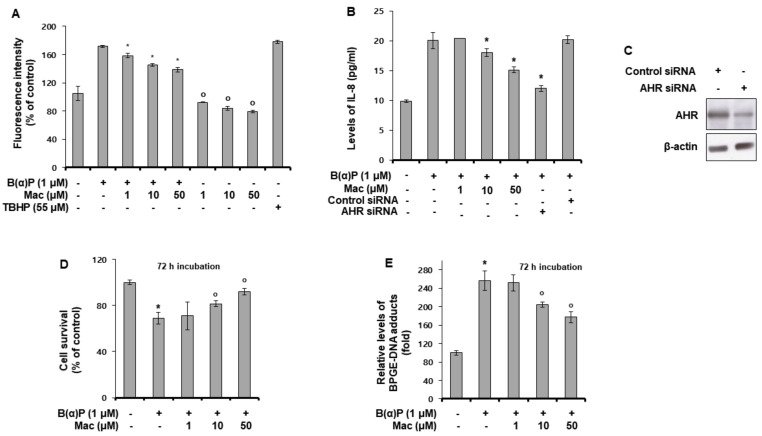
Maclurin attenuated intracellular production of ROS and IL-8, and reduced cell survival and BPDE-DNA adduct formation induced by B(a)P in HaCaT cells. (**A**) HaCaT cells were cultured with B(a)P and maclurin for 24 h, and the fluorescence intensity was analyzed. The data were verified by repeating independent experiment three times and the values represent the mean ± SD. * *p* < 0.05 vs. B(a)P treatment group, ^o^ *p*< 0.05 vs. untreated control. (**B**,**C**) HaCaT cells were transfected with AHR siRNA using the DharmaFECT^®^ Duo transfection reagent. After 24 h incubation, the cells were incubated with maclurin (1, 10, 50 μM) and B(a)P (1 μM) for 24 h and then analyzed by ELISA for IL-8 (**B**) and Western blotting for AHR (**C**). All results were confirmed from at least three independent experiments and the values represent the mean ± SD. * *p* < 0.05 vs. B(a)P treatment group. (**D**,**E**) HaCaT cells were treated with maclurin (1, 10, 50 μM) and B(a)P (1 μM) for 72 h, and the cell survival assay (**D**) and BPDE-DNA adduct formation analysis (**E**) were performed. The data are expressed as the mean ± S.D. * *p* < 0.05 vs. untreated control, ^o^ *p* < 0.05 vs. B(a)P-treated control. The data were confirmed from at least three independent experiments. TBHP: Tert-butyl hydroperoxide, Mac: maclurin.

During the metabolism of B[a]P, ROS is generated [27]. Therefore, the effects of maclurin on B(a)P-induced ROS production was examined. As shown in Figure 2A, maclurin reduced the ROS levels increased by B(a)P treatment as well as decreased the basal ROS generation in the absence of B(a)P (Figure 2A). Tert-butyl hydroperoxide (TBHP) was used as a positive control. IL-8 production induced by B(a)P was also decreased upon maclurin treatment (Figure 2B). The knock-down of the AHR gene using siRNA showed the same effect as maclurin treatment. AHR siRNA successfully knock-downed AHR protein in the HaCaT cells compared to the control siRNA (Figure 2C). This suggests that the maclurin effect on IL-8 production was mediated via the downregulation of the AHR gene. In addition, maclurin treatment attenuated the B(a)P effect on cell survival (Figure 2D) and BPDE-DNA adduct formation (Figure 2E). These data indicate that maclurin exerted antagonizing effects against B(a)P.

### 3.3. Maclurin Shows Radical Scavenging Property and Activates ARE Signaling

**Figure 3:**
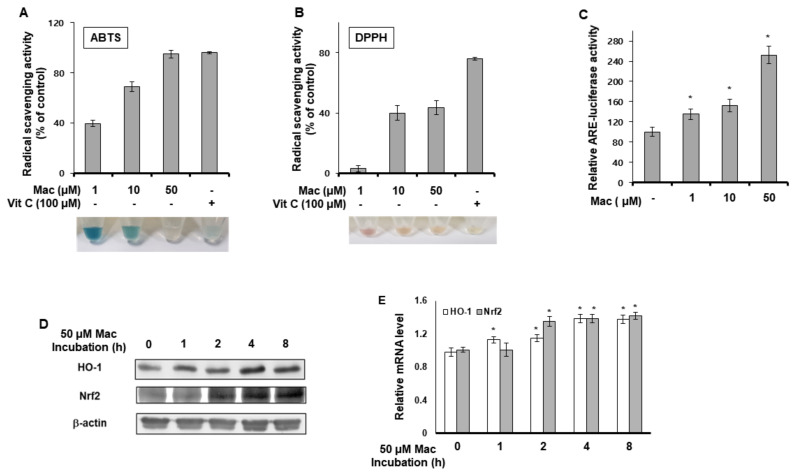
Maclurin showed radical scavenging property and activated ARE signaling. (**A**,**B**) ABTS (**A**) and DPPH (**B**) radical scavenging activity of maclurin. The results are shown as the mean ± SD of three replicates. (**C**) HaCaT cells were firstly transfected with the ARE-Luc reporter and a Renilla-luciferase vector using the DharmaFECT^®^ Duo transfection reagent. After incubation for 24 h, the cells were treated with maclurin for 14 h. The cells were lysed and subjected to the luciferase reporter assay. The data are shown as the mean ± SD of three replicates. * *p* < 0.05 vs. untreated control. (**D**,**E**) HaCaT cells were incubated with maclurin for the indicated time (1–8 h). The cells were harvested and subjected to Western blots and RT-PCR for HO-1 and Nrf2. The data are shown as the mean ± SD of three replicates. * *p* < 0.05 vs. untreated control. Vit C: vitamin C, Mac: maclurin.

We examined whether maclurin had radical-scavenging activity as an antioxidant using the ABTS and DPPH assays. As shown in Figure 3A,B, maclurin showed radical-scavenging activity, indicating that maclurin acted as an antioxidant. We also investigated the involvement of maclurin in ARE signaling. Maclurin enhanced the ARE reporter activity concentration-dependently (Figure 3C). In addition, maclurin treatment increased the protein (Figure 3D) and mRNA (Figure 3E) levels of Nrf2. Similarly, maclurin treatment increased the expression of HO-1, a target gene of Nrf2 (Figure 3D,E). These results indicate that maclurin can not only chemically act as an antioxidant with radical-scavenging activity, but can also biologically induce ARE signaling.

### 3.4. Maclurin Induces Nuclear Translocation of Nrf2 and Activates Nrf2-Dependent ARE Signaling

**Figure 4:**
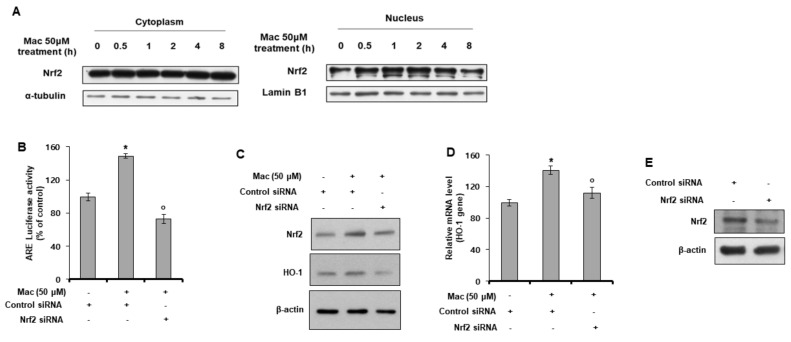
Maclurin induced nuclear translocation of Nrf2 and activated Nrf2-dependent ARE signaling. (**A**) HaCaT cells were treated with maclurin for 24 h and analyzed by Western blots of the cytosolic and nuclear fractions to assess Nrf2 translocation. (**B**) HaCaT cells were transfected with the ARE-Luc reporter and a Renilla-luciferase vector and siRNA for the Nrf2 gene using the DharmaFECT^®^ Duo transfection reagent. After 24 hincubation, the cells were treated with maclurin for 14 h. The cells were lysed and analyzed by the luciferase reporter assay. The data are presented as the mean ± SD of three replicates. * *p* < 0.05 vs. untreated control, ^o^ *p* < 0.05 vs. maclurin-treated control. (**C**–**E**) HaCaT cells were transfected with Nrf2 siRNA using the DharmaFECT^®^ Duo transfection reagent. After incubation for 24 h, the cells were treated with maclurin for 14 h. The cells were lysed and analyzed by Western blots (**C**,**E**) and RT-PCR (**D**). The data are presented as the mean ± SD of three replicates. * *p* < 0.05 vs. untreated control, ^o^ *p* < 0.05 vs. maclurin-treated control. Mac: maclurin.

In previous experiments, we found that maclurin activated ARE signaling. Next, we examined whether maclurin also affected the nuclear translocation of Nrf2. As shown in Figure 4A, maclurin increased the nuclear translocation of the Nrf2 protein. In the ARE-Luc reporter assay using siRNA for Nrf2, the maclurin-induced activation of ARE was reduced by the knock-down of the Nrf2 gene (Figure 4B), indicating that maclurin activated ARE signaling through Nrf2. These Nrf2-dependent effects of maclurin were also confirmed by Western blot analysis and RT-PCR analysis using siRNA for Nrf2. As shown in Figure 4C,D, while maclurin increased the protein and mRNA levels of Nrf2 and HO-1, knock-down of the Nrf2 gene attenuated the maclurin effect on the HO-1 protein and mRNA levels. Nrf2 siRNA successfully knock-downed AHR protein in HaCaT cells (Figure 4E). These findings indicate that the maclurin-induced ARE signaling was mediated by Nrf2.

### 3.5. p38 MAPK Activation Mediates Maclurin-Induced Nrf2 Activation

**Figure 5:**
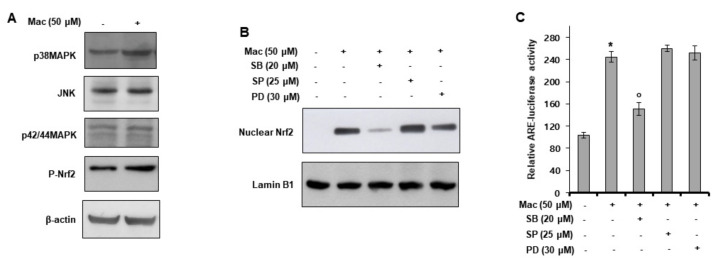
Maclurin-induced Nrf2 activation is mediated by p38 MAPK activation. (**A**) HaCaT cells were treated with maclurin for 1 h and analyzed by Western blots. (**B**) HaCaT cells were treated with maclurin and SB203580, SP600125, or PD98059 for 24 h and analyzed by Western blotting of the cytosolic and nuclear fractions to assess Nrf2 translocation. (**C**) HaCaT cells were transfected with the ARE-Luc reporter using the DharmaFECT^®^ Duo transfection reagent. After 24 h incubation, the cells were treated with maclurin in the presence of SB203580, SP600125, or PD98059 for 14 h. The cells were lysed and analyzed by the luciferase reporter assay. The data are shown as the mean ± SD of three replicates. * *p* < 0.05 vs. untreated control, ^o^ *p* < 0.05 vs. maclurin-treated control. Mac: maclurin.

We investigated the mechanism of action of maclurin in Nrf2 signaling. First, we performed a Western blot analysis of MAPKs and Nrf2. As shown in Figure 5A, maclurin induced the phosphorylation of Nrf2 and p38 MAPK, but not JNK and p42/44 MAPK. To examine the involvement of p38 MAPK in the nuclear translocation of Nrf2, we conducted Western blot analysis of the nuclear fraction. While SP600125 (a JNK inhibitor) or PD98059 (a p42/44 MAPK inhibitor) did not affect the maclurin-induced nuclear translocation of Nrf2, SB203580 (a p38 MAPK inhibitor) attenuated the maclurin effect (Figure 5B). Similar results were seen in the ARE-Luc reporter assay (Figure 5C). Namely, while SB203580 attenuated the maclurin-induced ARE activation, SP600125 and PD98059 showed no effect. These results indicate that maclurin activated Nrf2 signaling by activating p38 MAPK.

## 4. Discussion

In this study, we demonstrated the antagonizing effects of maclurin against B[a]P in human epidermal keratinocytes. Maclurin inhibited AHR signaling, as evidenced by the suppression XRE reporter activation, CYP1A1 expression, and ROS production induced by B[a]P in HaCaT cells. In addition, maclurin treatment activated Nrf2, as shown by activation ARE reporter and the enhanced expression of ARE-dependent genes. We demonstrated that the antagonist effects of maclurin on B[a]P were mediated by inhibiting AHR through the maintenance of the AHR-AIP complex and activating Nrf2 through p38 MAPK activation.

The skin acts as a protective barrier against the external environment. The skin consists of three parts, the epidermis, dermis, and hypodermis. Among them, the epidermis is the outermost part of the skin and is composed almost entirely of keratinocytes [28]. Due to the position of the keratinocytes in the skin, keratinocytes are the first cells to recognize and respond to the external environment. Therefore, in this study, we introduced a human keratinocyte cell line to examine the effects of maclurin on B[a]P-induced damage to the skin.

AHR is a xenobiotic chemical sensor protein, activated by various exogenous and endogenous ligand molecules [29]. The exogenous ligands include polycyclic aromatic pollutants, food metabolites, dioxins, B[a]P, and phytochemicals. Tryptophan photoproduct 6-formylindolo[3 ,2-b]carbazole is a UV irradiation-generated endogenous ligand with a high-affinity for AHR [30]. AHR activation induces the expression of cytochrome P450 1A1 (CYP1A1) which has been known as a member of the multigene family of xenobiotic-metabolizing enzymes. Although CYP1A1 contributes to detoxification [31], it can induce deleterious effects by producing ROS and mutagenic metabolites [32]. In this study, maclurin treatment attenuated the B[a]P-induced translocation of AhR to the nucleus. Maclurin reduced CYP1A1 expression and the production of ROS and IL-8 induced by B[a]P treatment. In addition, we demonstrated that while the B[a]P induced dissociation of the AHR-AIP complex, maclurin suppressed the dissociation induced by B[a]P. Therefore, these results suggest that maclurin suppressed the pro-inflammatory and oxidative stress effects induced by B[a]P by inhibiting the dissociation of the AHR-AIP complex.

Nrf2 is a transcription factor which regulates the expression of antioxidant genes [33,34]. Under normal conditions, Nrf2 interacts with Keap1 and CUL3, forming the Nrf2-Keap1-CUL3 complex [35]. Due to Nrf2-Keap1-CUL3 complex formation, Nrf2 remains in the cytoplasm. Increased levels of oxidative stress oxidize cysteine residues in Keap1 that change its conformation, resulting in the dissociation of the Nrf2-Keap1-CUL3 complex. In addition, at this stage, Nrf2 becomes phosphorylated by several molecules such as MAPKs and Akt [36,37]. Free Nrf2 then translocates to the nucleus and upregulates the expression of antioxidant genes [38]. In this study, maclurin treatment increased the nuclear translocation of Nrf2, and upregulated the expression of HO-1, a target gene of Nrf2. We also found that maclurin induced Nrf2 phosphorylation and its effect on Nrf2 phosphorylation was attenuated by SB203580 (a p38 MAPK inhibitor). These results suggest that maclurin induced Nrf2 signaling through p38 MAPK activation. In addition, B(a)P can induce deleterious effects by increasing the intracellular production of ROS and IL-8, decreasing cell survival, and increasing BPDE-DNA adduct formation [39,40]. In this study, maclurin treatment reduced these negative effects of B(a)P. These results indicate that maclurin protected the cells from oxidative stress by activating Nrf2 signaling through p38 MAPK phosphorylation.

It has been reported that Nrf2 is associated with skin diseases. Nrf2-null mice showed significantly stronger and longer-lasting sunburn reactions to UVB radiation [41]. Human malignant skin tumors have been reported to have lower expression levels of Nrf2 than normal cells [42]. Mutation of the NRF2 gene has also been found in some squamous cell carcinoma cases [43]. In addition, maclurin possesses anticancer activity. In this study, we demonstrated that maclurin was protective against B[a]P-induced damage to human epidermal keratinocytes with antioxidative effects. These effects of maclurin were mediated by inhibiting AHR signaling through maintenance of the AHR-AIP complex and activation of Nrf2 signaling via p38 MAPK. Our results suggest that maclurin could be used as an agent for improving abnormal skin physiologies such as sunburn and skin tumors, as well as skin symptoms induced by B[a]P. Specifically, maclurin could be developed in the form of pharmaceuticals or cosmeceuticals. However, experiments (pharmacokinetics and pharmacodynamics) using animals are needed to verify efficacy and safety of cotreatment with maclurin and B[a]P.

## 5. Conclusions

**Figure 6:**
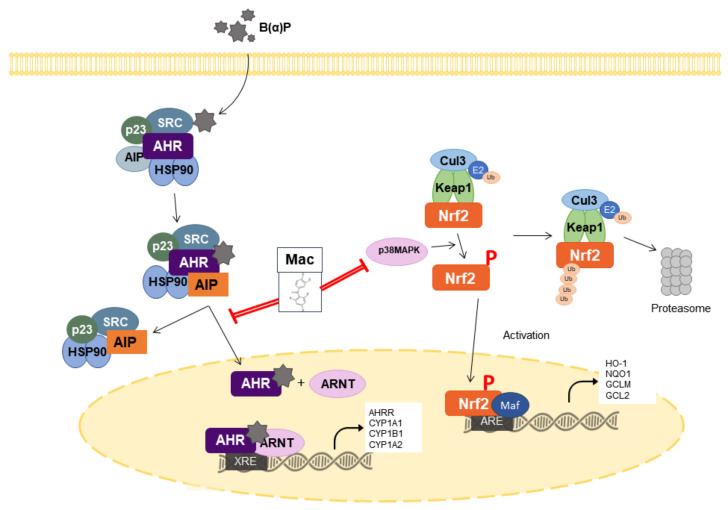
Action mechanism of maclurin in the AHR/Nrf2-mediated signaling. Maclurin inhibits B[a]P effects by inhibiting AHR through maintenance of the AHR-AIP complex and activating Nrf2 through p38 MAPK activation. Red line: Action step of maclurin.

This study demonstrated the antagonizing effects of maclurin against B[a]P in human epidermal keratinocytes. Maclurin inhibited AHR signaling, as evidenced by the suppression of XRE reporter activation, CYP1A1 expression, and ROS production induced by B[a]P in HaCaT cells. In addition, maclurin treatment activated Nrf2, as shown by ARE reporter activation, and increased the expression of ARE-dependent genes. This study demonstrated that the antagonist effects of maclurin on B[a]P were mediated by inhibiting AHR through maintenance of the AHR-AIP complex and activating Nrf2 through p38 MAPK activation, as summarized in Figure 6. These results suggest that maclurin could protect the stratum corneum layer of the epidermis from harmful B[a]P-containing pollutants.

## Data Availability

Data is contained within the article.
